# A Mobile Phone–Based Healthy Lifestyle Monitoring Tool for People With Mental Health Problems (MyHealthPA): Development and Pilot Testing

**DOI:** 10.2196/10228

**Published:** 2018-10-01

**Authors:** Louise Thornton, Frances Kay-Lambkin, Bree Tebbutt, Tanya L Hanstock, Amanda L Baker

**Affiliations:** 1 National Drug and Alcohol Research Centre University of New South Wales Randwick Australia; 2 Priority Research Centre for Brain and Mental Health University of Newcastle Callaghan Australia; 3 School of Psychology University of Newcastle Callaghan Australia; 4 School of Medicine and Public Health University of Newcastle Callaghan Australia

**Keywords:** telemedicine, mental health, cardiovascular diseases, mhealth, smartphone, mobile phone

## Abstract

**Background:**

People with mental health disorders live, on average, 20 years less than those without, often because of poor physical health including cardiovascular disease (CVD). Evidence-based interventions are required to reduce this lifespan gap.

**Objective:**

This study aimed to develop, test, and evaluate a mobile phone–based lifestyle program (MyHealthPA) to help people with mental health problems improve key health risk behaviors and reduce their risk of CVD.

**Methods:**

The development of MyHealthPA occurred in 3 stages: (1) scoping of the literature, (2) a survey (n=251) among people with and without the experience of mental health problems, and (3) program development informed by stages 1 and 2. A small pilot trial among young people with and without mental health disorders was also conducted. Participants completed a baseline assessment and were given access to the MyHealthPA program for a period of 8 weeks. They were then asked to complete an end-of-treatment assessment and a follow-up assessment 1 month later.

**Results:**

In the study, 28 young people aged 19 to 25 years were recruited to the pilot trial. Of these, 12 (12/28, 43%) had been previously diagnosed with a mental illness. Overall, 12 participants (12/28, 43%) completed the end-of-treatment assessment and 6 (6/28, 21%) completed the follow-up assessment. Small improvements in fruit and vegetable consumption, level of physical activity, alcohol use, and mood were found between baseline and end of treatment and follow-up, particularly among people with experience of mental health issues. Most participants (history of mental illness: 4/7, 57%; no history of mental illness: 3/5, 60%) reported the program had above average usability; however, only 29% (2/7, no history of mental illness) to 40% (2/5, history of mental illness) of participants reported that they would like to use the program frequently and would recommend it to other young people. Participants also identified a number of ways in which the program could be improved.

**Conclusions:**

This study describes the formative research and process of planning that formed the development of MyHealthPA and the evidence base underpinning the approach. The MyHealthPA program represents an innovative approach to CVD risk reduction among people with mental health problems. MyHealthPA appears to be an acceptable, easy-to-use, and potentially effective mHealth intervention to assist young people with mental illness to monitor risk factors for CVD. However, ways in which the program could be improved for future testing and dissemination were identified and discussed.

## Introduction

People with mental health disorders live on average 20 years less than the general population [1]. Cardiovascular disease (CVD) is the leading cause of this excess mortality and is responsible for more deaths in this population than suicide [2]. Smoking, alcohol misuse, physical inactivity, and poor diet are consistently identified as the top 4 behavioral risk factors associated with CVD in the general population [3]. These modifiable behavioral risk factors are over-represented among people with mental health problems [4]. These behaviors also commonly co-occur in clusters, which presents opportunities to adopt a multiple health behavior–change approach, in which behavioral risk factors are targeted together, rather than in isolation.

Recent research has shown that changing multiple behavioral risk factors and reducing CVD risk is possible among people with mental health problems [5]. However, because of time constraints and lack of awareness, training, and resources, mental health services often confine their services to mental health issues alone, neglecting physical health and CVD risk [6]. In addition, many people with mental health problems do not access treatment for their concerns, with Australian data indicating that only around one-third of people with mental health problems in the past 12 months accessed treatment [7]. There is a need to develop effective interventions to address CVD risk among this population and that are accessible both within and outside mainstream health and mental health services. It is also imperative that these interventions are scalable and of low burden to both clinicians and patients.

Mobile phone–based interventions to reduce CVD risk may be able to address these needs. Through mobile devices, individualized interventions can be provided inexpensively to a large number of people, including those who are geographically isolated, at a time and place when they are ready to engage in treatment [8]. Mobile phone–based interventions have been shown to be effective in improving a range of behavioral risk factors associated with CVD, including physical activity, weight loss, alcohol use, and smoking cessation, as well as mental health problems, including depression and anxiety [9]. They may also be especially useful in engaging people with mental health problems with treatment. In a recent systematic review, Donker et al [10] found adherence rates for smartphone apps targeting a range of mental health issues were high [10]. This study describes the development and initial evaluation of the first mobile phone–based monitoring tool to target multiple modifiable CVD behavioral risk factors (smoking, alcohol misuse, poor diet, and inadequate physical activity) for people with mental health problems (MyHealthPA).

## Methods

The development of MyHealthPA occurred in 3 stages: (1) scoping of the literature, (2) survey among people with and without experience of mental health problems, and (3) program development. These stages are detailed below.

### Stage 1: Scoping of the Literature

This stage aimed to identify, from previous research, the key strategies required to improve behavioral risk factors associated with CVD and develop a mobile phone–based tool for people with mental health problems. The main features considered in our examination of the existing literature included the intervention content and the delivery and design of the intervention.

#### Intervention Content

Ward et al [11] conducted a meta-review of nonpharmacological lifestyle interventions for CVD risk factors among the general population and those with severe mental disorders. All of the interventions they identified addressed behavior change related to diet or exercise and revealed a number of common factors for success in these interventions. This was a common observation among the existing literature identified (ie, a focus on diet and exercise and not tobacco or alcohol misuse, despite these being key behavioral risk factors for CVD and highly prevalent among people with mental health disorders). The review by Ward et al [11] reported that many successful interventions employed cognitive behavioral therapy (CBT) techniques (eg, goal setting, self-monitoring of food intake and physical activity, and the use of a structured curricula to encourage behavior change), with use of a greater number of strategies associated with improved results. Other key elements of successful interventions included the use of multiple components (eg, addressing diet and exercise and using CBT techniques), personalization of diet and exercise regimens, increased duration of the intervention, higher frequency of contact, and multidisciplinary teams. Of note was that face-to-face interventions (as opposed to computer-based interventions) were associated with better results in this meta-review. However, Ward et al [11] only included studies published before 2012 and did not include any mobile phone–based interventions. The field of technology-based behavior change interventions has grown considerably since that time, and thus, this particular observation may no longer be valid. Although fewer reviews of interventions among individuals with severe mental illness were identified, Ward et al [11] found that among this subpopulation, single component programs were also less effective than those employing multiple components. However, few interventions among this population actually employed multiple components. Manualized interventions were also rarely employed, group sessions rather than individually personalized interventions were more commonly used, and interventions were often of short duration.

Baker et al conducted the only trial to-date of interventions for people with mental health disorders targeting smoking and alcohol use as well as diet and exercise [5]. The trial compared an intensive face-to-face intervention with a brief telephone-based intervention, both of which used CBT and motivational interviewing techniques, including self-monitoring and goal setting, to encourage behavior change across multiple targets. In both conditions, there were significant improvements in smoking abstinence, cigarettes per day, and expired carbon monoxide at 15-week and 12-month follow-up. A significant reduction in 10-year CVD risk of participants was observed at 15-week follow-up, which continued to the 12-month follow-up for the brief telephone-based condition. In a subsequent trial by the same group, a telephone-based intervention targeting fruit and vegetable consumption, leisure screen time (a newer health behavior of increased research interest), and alcohol use was also associated with significant improvements in fruit and vegetable consumption, quality of life, and leisure screen time and sitting time (also a new health behavior receiving increasing research interest) [12] in people with mental health problems.

Key CBT techniques such as self-monitoring and goal setting have been identified as central to successful CVD risk–reduction interventions among people with and without mental health problems. Meta-analyses provide evidence for the efficacy of self-monitoring of diet, physical activity, weight, and tobacco and alcohol use [13-16]. The ability of patients to easily monitor their behaviors and moods, set goals, and track their progress through mobile phone–based interventions has also been identified as one of the many advantages of using mobile devices to deliver health and mental health interventions [10,17]. Electronic self-monitored mood has also been found to be valid compared with clinical rating scales of depression [18].

#### Delivery and Design of the Intervention

The available literature suggests that even the most popular existing mobile health apps (eg, MyFitnessPal) have poor usability, even among the general population [19]. People with mental health problems, particularly those with severe mental illnesses, can experience concomitant cognitive impairments, which may mean mobile phone–based interventions using standard design principles are less usable [8,20].

Rotondi et al [20] conducted a series of design and usability studies among people with severe mental illnesses to create the first empirically based design model for the development of eHealth tools for this population. Their Flat Explicit Design Model (FEDM) contains 18 design recommendations that aim to reduce the cognitive effort required to effectively use an eHealth tool and thus allow these tools to be usable by people with severe mental illnesses. This model recommends a *flat design* (with no more than 2 levels in the site structure of the website or the app), using descriptive labels and explicit instructions (as opposed to succinct, but often abstract labels or symbols) and using text written at a low reading level [20,21]. Employing this approach is argued to reduce the need for users to think abstractly, rely on working memory to create a mental model of the website or app, use executive functions to search for information or explore the website or app effectively, and concentrate to filter out distracting contents.

Ferron et al [21] conducted some of the only research to investigate the usability of publicly available mobile health apps among people with mental health problems. They investigated whether smoking cessation apps are usable by smokers with psychotic disorders. Overall, 21 smokers with a psychotic disorder assessed the usability of 9 smoking cessation apps (previously rated to be of high quality by expert reviewers). Their research identified multiple features of currently available smoking cessation apps that caused these apps to be inaccessible or ineffective among most smokers with psychotic disorders. These barriers included the use of text-heavy designs; difficulty navigating the apps because of the use of jargon and abstract symbols; and the use of subtle directions, such as the provision of only small symbols or 1-word instructions as cues how to use the apps [21].

### Stage 2: Survey Among People With and Without Experience of Mental Health Problems

To ensure the MyHealthPA program was tailored to the needs of people with mental health problems, scoping research with potential end users of the program was conducted.

#### Participants and Procedure

Participants were recruited via paid and unpaid advertisements on social media to participate in a brief survey of attitudes toward using mobile phone–based technology for health-related behaviors. Those who clicked on the study advertisements were directed to a Web-based information statement and consent form and then directed to the self-report questionnaire hosted by the Web-based survey program Fluid Surveys if they chose to participate. The survey was also sent to 200 members of a community research register who were asked to return the consent form and self-report questionnaire in a reply-paid envelope. Participants were required to be aged over 18 years and currently living in Australia.

#### Measures

The survey included items regarding demographic characteristics, mobile phone access and use, and openness to using mobile phone technologies for health purposes. Participants were also asked to indicate if they had ever been diagnosed with a mental illness. Current psychological distress was assessed using the Patient Health Questionnaire, PHQ 4 [22]. Participants indicated if they were a current smoker (yes or no) and their use of tobacco and alcohol in the past 3 months (never, once or twice, monthly, weekly, and daily or almost daily).

#### Findings

Of the 722 people who accessed the Web-based survey, 334 (334/722, 46.2%) provided consent and were eligible to participate in the study and 249 (249/722, 34.4%) provided sufficient data to be included in the analysis of this study. Of the 200 members of the community research register contacted, 35 (35/200, 17.5%) returned completed questionnaires. The final sample of 284 participants was aged between 18 and 77 years (mean 30.64, SD 14.49). The majority of participants were females (152/284, 53.5%), held a university degree (141/284, 49.6%), were employed (156/284, 54.9%), and lived in a major city (223/284, 78.5%). Approximately half of participants reported a history of mental illness (137/284, 48.2%), including 109 with a history of depression, 108 with a history of anxiety, 5 who had been previously diagnosed with a psychotic disorder, 11 with an eating disorder, 5 with a bipolar disorder, 8 with a borderline personality disorder, and 6 with a posttraumatic stress disorder. However, most (181/251, 72.1%) reported no (or mild) current psychological distress.

Of the 251 participants who provided information about their mental health status, 144 (144/251, 57.4%) reported experiencing mental health problems, including 61 (61/251, 24.3%) who reported a history of mental illness and current psychological distress, 73 (73/251, 29.1%) reporting a history of mental illness but no current psychological distress, and 10 (10/251, 4.0%) reporting current psychological distress but no history of mental illness. Overall, 107 participants (107/251, 42.6%) reported neither a history of mental illness nor current psychological distress.

Participants reported extremely high levels of access to mobile phone technology, with 93.5% to 100% of participants reporting they owned or had easy access to a mobile phone. The majority of participants had previously used their mobile phone to access information or treatment for physical health concerns (184/251, 73.3%). Most participants with a history of mental illness or current psychological distress had also done so specifically for mental health concerns (114/144, 79.2%). Fewer had accessed information or treatment for drug and alcohol concerns (52/144, 36.1%). Across these different types of health concerns, most participants reported that they would consider accessing treatment via a mobile phone (62.3%-75.8%).

When asked if they were interested in receiving information or treatment via a mobile phone about a range of health concerns, few participants (12.5%-29.1%) were interested in specifically addressing CVD (see Table 1). However, more participants did express interest in addressing key CVD behavioral risk factors. For example, most participants were interested in addressing physical activity (116/190, 61.1%) and diet (93/190, 49.0%) via mobile phone. Among those with mental health problems (ie, a history of mental illness or current psychological distress, n=111), the majority were also interested in addressing mood (60.0%-64.6%) and mental health issues (72.9%-87.5%).

Although very few participants, overall, were interested in addressing smoking (20/190, 10.5%) or alcohol use (24/190, 12.6%) via a mobile phone, interest in addressing these issues was higher among frequent users of these substances. Among participants reporting daily (or almost daily) use of alcohol, 60% (6/10) of participants with a mental health problem (Those with current distress, a history of mental illness or both) reported that they would be interested in addressing alcohol use via a mobile phone. Only 17% (2/12) of daily drinkers without a mental health problem were interested in addressing alcohol use via a mobile phone. Similarly, among daily smokers, 81% (13/16) of participants with mental health problems were interested in addressing smoking, and 75% (3/4) of smokers without a mental health problem were interested in addressing smoking.

### Stage 3: Program Development

The initial content of MyHealthPA was informed by the scoping of the literature and survey research described above. This study suggested it is appropriate to address risk of CVD for people with mental health problems using a mobile phone–based intervention. It also highlighted that the MyHealthPA program needed to, at a minimum, include self-monitoring and goal-setting techniques, provide feedback on behaviors of the users, adopt a multiple health behavior change framework, and should address individual risk factors as opposed to CVD specifically. The initial content was written so that it was brief, there was minimal introductory content, it explicitly communicated concepts, and it was easy to read, in line with the principles of the FEDM [20].

Furthermore, 2 academics and 2 clinicians with expertise in health behavior change among people with mental health problems reviewed the initial written content. Feedback on the initial content was that it was accurate and correct in accordance with the most current research and behavior change techniques. Any information that was queried was checked with the literature and changed accordingly. Minor changes to the language to improve the readability of content were also made.

A beta version of the MyHealthPA program was then developed. Initial usability testing was first undertaken, and any technical issues identified were resolved before the app was reviewed by 2 academics, 2 clinicians, and 2 mental health consumers. These reviewers provided feedback regarding the final content, usability, and appeal of the program. The beta version of MyHealthPA was informed by the FEDM [20]. For example, the program was designed so that it had a shallow hierarchy; therefore, to access the majority of features of the program, users need to go only 1 level past the initial home screen and only 2 levels for a minimal number of features. A simple pop-up menu bar at the top left of the home screen was used to facilitate navigation and comprehension, and a relatively plain visual design was employed to reduce distractions for users. During the beta feedback phase, reviewers were asked to rate their mood using a set of *emoticons* representing different ways they might be feeling (eg, happy, calm, tired, lonely, sad, angry, and depressed) to determine the validity of using these images and labels to represent mood.

The key change made to the program based on feedback from reviewers was that the emoticon mood-rating system was changed to a 10-point Likert scale where users answer the question “How do you feel today?” Descriptors of 1 = the worst I have ever felt of could ever imagine feeling; 5 = in the middle, neither very bad or very good; and 10 = the best I have ever felt or could ever imagine feeling were used to help guide users’ responses. If users select *1* on this scale, they are automatically presented with a pop-up message asking if they are ok, provided with the contact details for emergency helplines (eg, Lifeline and Kids helpline), and instructed to call emergency services if life is in danger or they are in need of emergency assistance.

#### The MyHealthPA Program

**Figure 1:**
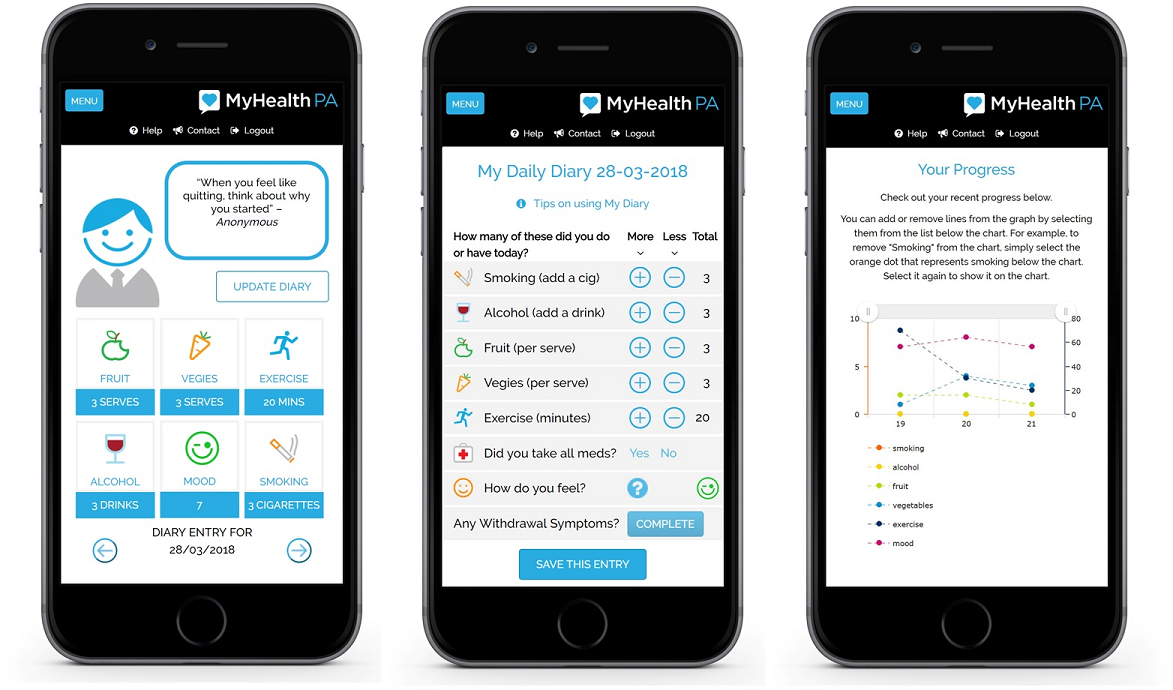
Screenshots of the MyHealthPA program.

MyHealthPA provides users with feedback regarding smoking, alcohol use, fruit and vegetable consumption, and physical activity; allows users to easily record their health behaviors and mood on any mobile device; and track their progress over time. Users can also set health behavior goals, and reminders are sent to record behaviors (see Figure 1).

When users first access MyHealthPA, they are asked to complete a brief questionnaire regarding their health behavior and mood, at the end of which they are provided with personalized feedback regarding their health behaviors based on national guidelines [23-25]. Users can then access the full MyHealthPA program, which consists of the following 7 pages or sections:

1. Home page: This page provides a simple and visual portrayal of the diary entry of the current day, a motivational quote from the Personal Assistant avatar, and a menu to access all other pages.
2. My Diary: This page allows users to record their mood and health behaviors (number of cigarettes, number of alcoholic drinks, minutes of physical activity, and/or serves of fruits and vegetables consumed) for the day. Participants can also record if they have taken any medications as prescribed that day and any withdrawal (scale name) or adverse psychiatric symptoms (scale name) they may have experienced.
3. My Progress: This page allows users to view their progress via an interactive graph that users can use to display changes in multiple health behaviors and/or mood over time.
4. My Goals: This page allows users to set goals, including due dates for these goals, related to each of the measured health behaviors (eg, set a quit date and quit smoking, reduce the number of alcoholic drinks in a day to XX, eat 2 servings of fruit per day, and exercise XX times per week) and displays any current goals they have set. A pop-up textbox also provides users with tips on setting Specific, Measurable, Active, Realistic, and Time-limited (SMART) goals.
5. My Profile: On this page, users can enter and edit their personal information (eg, name, gender, height, weight, contact details, and any medications they are taking) and customize the notifications they receive from the program.
6. Resources: This page provides links to Web-based resources that contain extra information and tips about changing health risk behaviors.
7. Emergency: This page provides contact details for relevant helplines. Participants are instructed to contact one of these services or contact emergency services if they are thinking about suicide or experiencing a personal crisis.

MyHealthPA was developed as a responsive website (as opposed to a native and downloadable app) optimized for use on a mobile phone, but that also allowed users to view the program on any device with internet access.

**Table 1:** Interest in addressing specific health issues via a mobile phone.

Health issue	History of mental illness, current distress^a^ (N=48), n (%)	History of mental illness, no distress (N=55), n (%)	No mental illness, current distress (N=8), n (%)	No mental illness, no distress (N=79) n (%)
Diet	23 (48)	30 (55)	3 (38)	37 (47)
Physical activity	29 (60)	37 (67)	5 (63)	45 (57)
Cardiovascular disorder	7 (15)	16 (29)	1 (13)	16 (20)
Smoking	9 (19)	6 (11)	1 (13)	4 (5)
Alcohol use	7 (15)	12 (22)	1 (13)	4 (5)
Mood	31 (65)	33 (60)	5 (63)	18 (23)
Mental health	35 (73)	41 (75)	7 (88)	30 (38)

^a^Distress: psychological distress.

#### Pilot Testing

To evaluate the feasibility and potential efficacy of MyHealthPA, particularly among people with mental health problems, a pilot study was conducted among young people with and without a previous diagnosis of a mental illness. The pilot study used a pre-post design. Participants were recruited via flyers placed on university campuses, paid and unpaid advertisements on social media (eg, Facebook or Twitter), and on the institution website of the lead author.

Potential participants were asked to complete an initial Web-based screening questionnaire. To be eligible, participants were required to be aged 18 to 25 years, live in Australia, and have access to a mobile phone with internet access. Upon meeting eligibility criteria, participants were asked to provide informed consent and complete a Web-based baseline assessment. They were then given access to the MyHealthPA program for a period of 8 weeks, after which they were asked to complete a Web-based end-of-treatment assessment and a Web-based follow-up assessment 1 month later (12 weeks after baseline). All Web-based assessments were hosted by Survey Monkey.

#### Measures

The baseline assessment contained items regarding demographic characteristics; medical history, including if they had ever been diagnosed with a mental illness; frequency of mobile phone use; use of mobile health apps; current health behaviors; and current psychological distress (using the 4-item version of the PHQ 4) [22].

The health behaviors measured were smoking (smoking status and cigarettes per day), alcohol use (Alcohol Use Disorders Identification Test—Consumption items, AUDIT-C [26], a brief measure of hazardous and harmful alcohol use), diet (serves of fruits and vegetables consumed per day), and physical activity (International Physical Activity Questionnaire [27]). This information was then used to calculate participants’ Lifestyle Risk Index (LRI) [28]. The LRI is a composite score, which represents compliance with national guidelines for the 4 health behaviors. Each behavior is assigned a score of *0* (compliance with guidelines, *not at-risk*) or *1* (noncompliance with guidelines, *at-risk*). Scores are then summed for an overall LRI score. The LRI method has been previously validated by Ding et al [28] in a large cohort of Australian adults. Furthermore, such an approach has been recommended for generating quantifiable outcomes in interventions for multiple risk factors [29].

Health behaviors and current psychological distress of participants were also assessed at end of treatment and follow-up. In addition, as a part of the end-of-treatment assessment, participants were asked to answer a series of questions related to the usability and acceptability of MyHealthPA, which included the System Usability Scale (SUS) [30], and open-ended questions regarding which sections of the program participants’ felt worked well or did not work well and any suggestions to improve the program.

## Results

### Overview

A total of 102 participants completed the initial Web-based screening questionnaire. Of these, 35 (35/102, 34.3%) were eligible to participate and provided informed consent; however, 7 (7/35, 20%) did not complete the Web-based baseline questionnaire, leaving a total of 28 (28/35, 80%) participants who were included in the pilot study and granted access to the MyHealthPA program. A total of 12 (12/28, 43%) participants also completed the end-of-treatment Web-based assessment, and 6 (6/28, 21%) participants completed the follow-up assessment 1 month later.

### Participant Characteristics

A total of 12 (12/28, 43%) participants reported that they had previously been diagnosed with a mental illness. Descriptive statistics are reported separately for participants with and without a history of mental illness. Of these, 9 (9/12, 75%) participants reported having previously been diagnosed with depression, 9 (9/12, 75%) with anxiety, 2 (2/12, 17%) with an eating disorder, 2 (2/12, 17%) with a bipolar disorder, and 1 (1/12, 8%) with a borderline personality disorder. As shown in Table 2, the majority of participants in both groups were females. Participants had a mean age of 21 years, most were single and had never been married, were born in Australia, and had a high level of education. Overall, 7 (7/28, 25%) participants identified as belonging to the lesbian, gay, bisexual, and transgender community (including half of the participants with a history of mental illness).

Both groups of participants described frequent mobile phone use. All participants used their mobile phone every day, and most of them used it at least once every hour (history of mental illness = 9/12, 75%, no history of mental illness = 12/16, 75%). Most participants (history of mental illness = 11/12, 92%, no history of mental illness = 12/16, 79%) had also previously used their mobile phone to look for health or medical information or track health and fitness data, with many reporting they did so on a weekly or daily basis (history of mental illness = 6/12, 50%, no history of mental illness = 5/16, 31%). The majority of participants also reported having a range of health apps installed on their mobile phone, particularly exercise (history of mental illness = 8/12, 67%, no history of mental illness = 9/16, 56%), diet (history of mental illness = 11/12, 92%, no history of mental illness = 14/16, 88%), sleep (history of mental illness = 67%, no history of mental illness = 31%), and mood apps (history of mental illness = 50%, no history of mental illness = 13%). However, most participants with these apps installed on their mobile phone reported rarely using them (history of mental illness = 5/12, 42% to 12/12, 100%, no history of mental illness = 11/16, 69% to 16/16, 100%).

**Table 2:** Participant characteristics at baseline.

Characteristics		History of mental illness (N=12)	No history of mental illness (N=16)
Age (years), range		19-25	18-25
Age (years), mean (SD)		21.2 (2.1)	21.81 (2.3)
Gender (female), n (%)		10 (83)	10 (63)
Lesbian, gay, bisexual, and transgender, and intersex, n (%)		6 (50)	1 (6)
ATSI^a^, n (%)		0 (0)	0 (0)
**Marital status, n (%)**			
	Defacto	1 (8)	1 (6)
	Never married or single	11 (92)	15 (94)
Born in Australia, n (%)		9 (75)	14 (88)
First language other than English, n (%)		1 (8)	3 (19)
**Highest education, n (%)**			
	High school (Grade 11-12)	9 (75)	9 (56)
	University degree	3 (25)	7 (44)
**Employment status, n (%)**			
	Employed (full or part time and casual)	2 (17)	4 (25)
	Student	9 (75)	9 (56)
	Unemployed	0 (0)	1 (6)
	Other	1 (8)	2 (13)
**Health risk behaviors, n (%)**			
	At risk—alcohol	5 (46)	5 (33)
	At risk—smoking	1 (14)	2 (13)
	At risk—diet	12 (100)	15 (100)
	At risk—physical activity	5 (46)	6 (40)
Lifestyle Risk Index, mean (SD)		1.90 (0.57)	1.86 (0.92)
PHQ 4^b^, mean (SD)		5.67 (2.96)	2.60 (2.07)

^a^ATSI: Aboriginal and Torres Strait Islander.

^b^PHQ 4: 4-item Patient Health Questionnaire.

### Use of MyHealthPA

**Figure 2:**
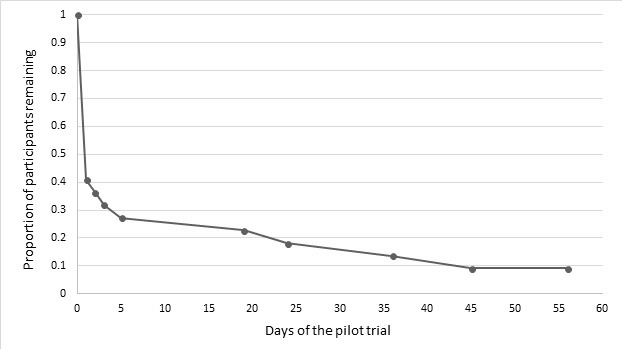
Attrition among MyHealthPA users.

As can be seen in Table 3, use of the MyHealthPA program varied widely among participants. Overall, 4 (4/12, 33%) participants with a history of mental illness and 2 participants without a history of mental illness (2/16, 13%) never accessed the program. Out of a possible 56 days, participants accessed MyHealthPA on a mean of 3.82 days. As can be seen in Figure 2, among the 22 participants who accessed MyHealthPA, most stopped accessing it after 1 day of use. Only 41% of participants continued to use the program past this point. However, past day 5, remaining participants went on to be active users of MyHealthPA for between 3 weeks and the full 8 weeks of the study.

### Usability and Acceptability

The 12 participants who completed the end-of-treatment assessment reported that MyHealthPA had average usability. The mean SUS scores were 67.1 among participants without a history of mental illness and 64.5 among those with a history of mental illness, with a slight majority (history of mental illness = 4/7, 57%, no history of mental illness = 3/5, 60%) reporting the MyHealthPA program had above-average usability (as indicated by a score of 68 or more on the SUS [30]). Specifically, as can be seen in Table 4, although only 29% (2/7) of participants without a history of mental illness and 40% (2/5) of participants with a history of mental illness agreed or strongly agreed that they would like to use the MyHealthPA program frequently, most participants agreed or strongly agreed that the program was easy to use and thought the program was consistent. Similarly, most participants disagreed or strongly disagreed that they would need the support of a technical person to use MyHealthPA and that they needed to learn many things before they could get going with the program. In addition, 43% (3/7) of participants without a history of mental illness and 60% (3/5) of participants with a history of mental illness thought MyHealthPA would help people to change their lifestyle behaviors. The majority thought the content of MyHealthPA was relevant to young people (history of mental illness = 4/5, 80%, no history of mental illness = 5/7, 71%), but only 29% (2/7) of participants without a history of mental illness and 40% (2/5) of those with a history of mental illness would recommend MyHealthPA to other young people.

When participants were asked what aspects of MyHealthPA they felt did not work well, a key theme of *difficulty accessing the program* emerged. Participants mentioned that the need to access the program via a Web browser on their mobile phone rather than simply opening a native app was a barrier to use, saying it “involved opening too many windows and logging in constantly.”

Similarly, participants found it difficult to remember to access the program regularly:

I found it difficult to remember to use it every day—in fact I completely forgot about it until I got the email to do this survey. A phone app with daily reminders would be a good idea.

The length of the adverse symptoms questionnaire was also cited as a barrier to use, and 1 participant without a history of mental illness questioned the simplicity of the program: “Maybe a bit TOO simple—didn’t really see the point in using it.”

**Table 3:** Participants’ use of MyHealthPA.

MyHealthPA use		Mean (SD)		Range	
**History of mental illness (N=12)**					
	Number of days accessed MyHealthPA		3.17 (8.47)		0-30
	Number of times access MyHealthPA		6.42 (13.69)		0-39
	Number of pages access		5.0 (11.49)		0-41
	Number of diary entries		9.50 (20.59)		0-54
	Number of goals set		0.25 (0.62)		0-2
**No history of mental illness (N=16)**					
	Number of days accessed MyHealthPA		4.31 (6.75)		0-24
	Number of times access MyHealthPA		7.13 (11.79)		0-38
	Number of pages access		11.25 (14.59)		0-44
	Number of diary entries		10.0 (16.56)		0-54
	Number of goals set		0.75 (1.07)		0-3

**Table 4:** System Usability Scale scores.

System usability scale		History of mental illness (N=5), n (%)	No history of mental illness (N=7), n (%)
**I think that I would like to use the MyHealthPA app frequently**			
	Strongly disagree	1 (20)	0 (0)
	Disagree	1 (20)	3 (43)
	Neutral	1 (20)	2 (29)
	Agree	1 (20)	2 (29)
	Strongly agree	1 (20)	0 (0)
**I found the MyHealthPA app unnecessarily complex**			
	Strongly disagree	2 (40)	0 (0)
	Disagree	2 (40)	3 (43)
	Neutral	1 (20)	2 (29)
	Agree	0 (0)	2 (29)
	Strongly agree	0 (0)	0 (0)
**I thought the MyHealthPA app was easy to use**			
	Strongly disagree	0 (0)	0 (0)
	Disagree	0 (0)	0 (0)
	Neutral	2 (40)	3 (43)
	Agree	3 (60)	3 (43)
	Strongly agree	0 (0)	1 (14)
**I think that I would need the support of a technical person to be able to use the MyHealthPA**			
	Strongly disagree	1 (20)	4 (57)
	Disagree	3 (60)	1 (14)
	Neutral	0 (0)	2 (29)
	Agree	0 (0)	0 (0)
	Strongly agree	0 (0)	0 (0)
**I found the various functions in the MyHealthPA app were well integrated**			
	Strongly disagree	0 (0)	0 (0)
	Disagree	1 (20)	1 (14)
	Neutral	1 (20)	2 (29)
	Agree	1 (20)	4 (57)
	Strongly agree	0 (0)	0 (0)
**I thought there was too much inconsistency in the MyHealthPA app**			
	Strongly disagree	0 (0)	2 (29)
	Disagree	4 (80)	1 (14)
	Neutral	1 (20)	4 (57)
	Agree	0 (0)	0 (0)
	Strongly agree	0 (0)	0 (0)
**I would imagine that most people would learn to use the MyHealthPA app very quickly**			
	Strongly disagree	0 (0)	0 (0)
	Disagree	0 (0)	0 (0)
	Neutral	3 (60)	1 (14)
	Agree	2 (40)	3 (43)
	Strongly agree	0 (0)	3 (43)
**I found the MyHealthPA app cumbersome to use**			
	Strongly disagree	0 (0)	2 (29)
	Disagree	2 (40)	1 (14)
	Neutral	1 (20)	3 (43)
	Agree	1 (20)	1 (14)
	Strongly agree	1 (20)	0 (0)
**I felt very confident using the MyHealthPA app**			
	Strongly disagree	0 (0)	0 (0)
	Disagree	0 (0)	1 (14)
	Neutral	3 (60)	2 (29)
	Agree	1 (20)	2 (29)
	Strongly agree	1 (20)	2 (29)
**I needed to learn a lot of things before I could get going with the MyHealthPA app**			
	Strongly disagree	1 (20)	3 (43)
	Disagree	3 (60)	2 (29)
	Neutral	1 (20)	2 (29)
	Agree	0 (0)	0 (0)
	Strongly agree	0 (0)	0 (0)

When asked what they thought worked well about the program, the primary theme mentioned by participants was simplicity and ease of use of MyHealthPA. Participants liked the simple interface, how easy the program was to use, and how quickly users could enter their information, saying, “It’s easy to use, it works well on mobile, and doesn’t take much time.”

Participants also enjoyed being able to track and view their health behaviors and mood and how they interacted over time as highlighted by the following participant with a history of mental illness:

Could easily track my progress and see how my lifestyle had changed. It also made me aware of what I was eating, because I didn’t eat many vegetables or fruit before, but when I wrote it down I became aware of how unhealthy my lifestyle was. I found it interesting that when I started eating healthier and exercising a little bit more, my mood increased quite dramatically.

Finally, the key changes to the MyHealthPA program recommended by the participants were converting the program to a native app format and allowing continual log-in. Other suggestions included adding a calendar view of diary entries, allowing information from other health tracking apps to be integrated into MyHealthPA, providing more (but customizable) reminders to use the program, and providing extra information such as recipe and exercise ideas.

### Health Behavior Change

As can be seen in Table 5, the greatest improvements were observed in fruit and vegetable consumption and physical activity of participants, where both groups reported improvements in these behaviors between baseline and end of treatment and baseline and follow-up. Some improvement in alcohol use among participants with a history of mental illness was observed, particularly between baseline and follow-up; however, participants with no history of mental illness actually reported a slight increase in the harmfulness of their alcohol use at both time points. Similarly, although no change in the number of cigarettes smoked per day was reported between baseline and end of treatment among participants with a history of mental illness, a slight increase at follow-up and among participants without a history of mental illness was reported. Participants with a history of mental illness reported improvement in psychological distress (as measured by the PHQ 4) at end of treatment and follow-up.

Participants with a history of mental illness also maintained their LRI score at end of treatment and improved it by follow-up. On the other hand, a slight increase in mean LRI among people without a history of mental illness was observed at end of treatment. Specifically, as can be seen in Table 6, one participant without a history of mental illness was no longer at risk for 1 behavior at end of treatment, whereas 1 had increased his or her risk behaviors by 2 at end of treatment. By follow-up, 3 participants with a history of mental illness were no longer at risk for 1 behavior they had previously been at risk for, 1 maintained his or her level of risk, and 1 increased his or her risk by 1 behavior. The remaining participants without a history of mental illness reported maintaining their level of risk between baseline and follow-up.

**Table 5:** Change in health behavior and mood outcomes between baseline, end of treatment, and follow-up.

Outcome measures	Baseline to end of treatment, mean change (SD)		Baseline to follow-up, mean change (SD)	
	History of mental illness (N=5)	No history of mental illness (N=7)	History of mental illness (N=5)	No history of mental illness (N=1)
AUDIT-C^a^ score	0.00 (1.73)^b^	2.14 (1.86)	−1.00 (1.73)^b^	4.0 (N/A^c^)
No. of cigarettes per day	0.00 (0.00)^b^	1.50 (2.12)	0.33 (0.58)	N/A
No. of fruit and vegetables per day	0.50 (1.00)^b^	0.71 (1.38)^b^	1.0 (1.58)^b^	3.0 (N/A)^b^
IPAQ^d^ score	864.60 (1104.83)^b^	745.5 (1555.13)^b^	1518.20 (1162.87)^b^	1523.5 (N/A)^b^
PHQ 4^e^	−1.4 (4.51)^b^	0.29 (3.73)	−2.4 (2.88)^b^	0 (N/A)^b^
LRI^f^	0.00 (0.00)^b^	0.14 (0.89)	−0.40 (0.89)^b^	0.00 (N/A)^b^

^a^AUDIT-C: Alcohol Use Disorders Identification Test–consumption items.

^b^Change in desired direction or no change.

^c^N/A: not applicable.

^d^IPAQ: International Physical Activity Questionnaire.

^e^PHQ 4: 4-item Patient Health Questionnaire.

^f^LRI: Lifestyle Risk Index.

**Table 6:** Change in Lifestyle Risk Index.

Change in risk behaviors	Baseline to end of treatment		Baseline to follow-up	
	History of mental illness (N=5), n (%)	No history of mental illness (N=7), n (%)	History of mental illness (N=5), n (%)	No history of mental illness (N=1), n (%)
Change of −1	0 (0)	1 (14)	3 (60)	0 (0)
No change	5 (100)	5 (71)	1 (20)	1 (100)
Change of +1	0 (0)	0 (0)	1 (20)	0 (0)
Change of + 2	0 (0)	1 (14)	0 (0)	0 (0)

## Discussion

### Principal Findings

The results of the initial pilot study of the MyHealthPA program suggest that MyHealthPA is an acceptable, easy-to-use tool that may help people to reduce key health risk behaviors associated with CVD, especially people with mental health problems. Although only a slight majority of participants thought the program had above-average usability, most participants described it as easy to use. Unlike previous literature that has found difficulty using diary features to be a common criticism of health apps [31], participants in this study reported that the MyHealthPA diary feature was simple and quick to use. It is unclear why people with a history of mental illness have reported more positive health behavior changes than people without a history of mental illness. Potentially, these results indicate that although the FEDM may mean eHealth tools designed using this model are more acceptable and effective among people with mental health problems, it may result in tools that are perceived to be too simple and are less effective among people without mental health problems. Promisingly, participants with a history of mental illness also did not report increases in their psychological distress over the study period.

### Limitations

The pilot study had a number of limitations, including the use of self-report measures only, which meant that participant characteristics and results were unable to be independently validated. As such, these results should be interpreted with a degree of caution. Another key limitation was the high rate of participant dropout between the baseline, end-of-treatment, and follow-up assessments. Participants did not receive any incentives or compensation for completing each of the assessment points beyond receiving an extra entry into a draw to win an iPad. In previous research conducted by the research team that has achieved much higher follow-up rates, an incentive of Aus $20 to Aus $50 per assessment has been offered to participants. Unfortunately, resource limitations meant that similar incentives were unable to be offered in this pilot study. This lack of incentive may have been responsible for the low follow-up rates observed, highlighting the potential importance of incentives or compensation for participation in this kind of research.

In addition, despite receiving reminders to access the program after 2 and 5 days of inactivity, large proportion of participants never accessed MyHealthPA or accessed the program on only a few occasions. For example, out of a possible 56 days on which participants could have accessed the program, the maximum number of days the program was accessed was 30, with a mean of just under 4 days. In this way, their minimal use mirrored their SUS responses, which indicated few participants would like to use MyHealthPA frequently, as well as their reported patterns of use of other health apps and other mobile health trials [32]. As highlighted by participants, this lower-than-desired use of the program may have been influenced by the responsive website platform of the program. It is anticipated that converting the MyHealthPA program to a native app format may help to increase the frequency with which users want to access MyHealthPA. Other strategies to increase the use may include building rewards into the program where users could earn stars or badges for recording behaviors or meeting set goals. Similarly, it has been suggested that greater tailoring or personalization in mobile health apps may promote greater use and engagement [32]; however, more work is needed to determine the best ways to encourage frequent use of health apps over an extended period.

Finally, these results may need to be interpreted with caution, as the participants recruited to this pilot study may not be representative of the wider population of people with experience of mental health problems. Participants with a history of mental illness in this pilot were highly educated and mostly studying or employed. Despite these limitations, these initial results are promising. Further testing of the efficacy of the MyHealthPA program, including determining the optimal way to integrate this program into existing clinical and public health care, is warranted.

### Conclusions

The aim of this study was to describe the formative research and process of planning that formed the development of the MyHealthPA program. MyHealthPA was developed to address the need for scalable and effective interventions to address the risk of CVD among people with mental health problems that are of low burden to both clinicians and consumers. MyHealthPA is unique, as it targets the top 4 behavioral risk factors associated with CVD (smoking, alcohol misuse, physical inactivity, and poor diet), which are also extremely common among people with mental health problems, while also addressing mood and the way in which mood and psychiatric symptoms might interact with these health behaviors. Although many apps purporting to help users improve their health have been developed for use by the general population, MyHealthPA is the first to specifically target people with mental health problems and aims to help them improve their health behaviors and decrease their cardiovascular risk. The program was designed to employ evidence-based techniques, such as self-monitoring, goal setting, and addressing multiple health behaviors simultaneously [11]. It was also designed to overcome some of the potential obstacles to the use of mobile health tools designed for the general population among people with mental health problems by adopting Rotondi et al’s FEDM [20]. In addition, the mobile phone–based platform of the program signifies that MyHealthPA could drastically extend the reach and scalability of CVD risk reduction programs for people with mental health problems. It is hoped that subject to further testing in fully powered trials and conversion to a native app format, MyHealthPA could be accessed directly by people with mental health problems who want to improve their health and/or used by health professionals to engage their patients with mental health problems with the treatment of their physical health and prevention of CVD. This program could be particularly useful for consumers and health professionals in rural or remote communities where access to other treatment options is limited or in situations where waiting periods before and between appointments are particularly lengthy.

The design process employed to develop MyHealthPA was time and resource efficient. A key strength was the inclusion of a range of perspectives (ie, expert researchers, clinicians, and potential end users) in the design process via the scoping survey and review of the of the written content and beta version of the app. Additional focus or laboratory-based testing (eg, using a think-aloud protocol [33] to gain a better understanding of how end users might navigate to and through the program) may have meant that the issues with the website delivery method were identified earlier in the design process. However, given the resource constraints of this particular project, using this simplified design process and a responsive website, as opposed to a native app, meant that a product could be developed and initial feasibility and effectiveness testing could be conducted. This allowed this study to show for the first time that using a mobile phone–based program to help people with mental health problems improve their health risk behaviors and reduce their CVD risk may be feasible and effective.

Overall, the MyHealthPA program represents an innovative approach to CVD risk reduction among people with mental health problems. It appears that MyHealthPA is acceptable, easy to use, and potentially effective. A large-scale clinical trial employing MyHealthPA in groups of people with mental health problems is indicated.

## Abbreviations

AUDIT-C: Alcohol Use Disorders Identification Test–consumption items
CBT: cognitive behavioral therapy
CVD: cardiovascular disease
FEDM: Flat Explicit Design Model
SMART: Specific, Measurable, Active, Realistic, and Time-limited
SUS: System Usability Scale
LRI: Lifestyle Risk Index
PHQ 4: 4-item Patient Health Questionnaire
